# Socioeconomic costs and health-related quality of life associated with oculopharyngeal muscular dystrophy

**DOI:** 10.3389/fpubh.2026.1918801

**Published:** 2026-10-12

**Authors:** Ian C. Smith, Alyssa Grant, Lola E. R. Lessard, Homira Osman, Fien Oelbrandt, Hanns Lochmüller, Hugh J. McMillan, Gerald Pfeffer, Lawrence Korngut, Cynthia Gagnon, Stacey Lintern, Katherine A. Selby, Ari Breiner, Kednapa Thavorn, Jodi Warman-Chardon

**Affiliations:** 1 The Ottawa Hospital Research Institute, Ottawa, ON, Canada; 2 School of Epidemiology and Public Health, University of Ottawa, Ottawa, ON, Canada; 3 Université Claude Bernard Lyon Est, Lyon, France; 4 Hôpital Neurologique de Lyon, Hospices Civils de Lyon, Lyon, France; 5 PGNM, Institut Neuromyogène, CNRS UMR 5261 - INSERM U1315, Lyon, France; 6 Faculty of Medicine, University of Ottawa, Ottawa, ON, Canada; 7 Muscular Dystrophy Canada, Toronto, ON, Canada; 8 Department of Medicine (Neurology), The Ottawa Hospital/University of Ottawa, Ottawa, ON, Canada; 9 Children’s Hospital of Eastern Ontario, Ottawa, ON, Canada; 10 Department of Clinical Neurosciences, Cumming School of Medicine, Hotchkiss Brain Institute, University of Calgary, Calgary, AB, Canada; 11 Department of Medical Genetics, Cumming School of Medicine, Alberta Child Health Research Institute, University of Calgary, Calgary, AB, Canada; 12 Faculty of Medicine and Health Sciences, University of Sherbrooke, Sherbrooke, QC, Canada; 13 British Columbia Children’s Hospital, Vancouver, BC, Canada

**Keywords:** cost of illness, cross-sectional survey, economic burden, health economics, health utility, health-related quality of life, neuromuscular disease, patient-reported outcomes

## Abstract

**Background:**

Oculopharyngeal muscular dystrophy (OPMD) is a progressive genetic neuromuscular disease. OPMD substantially impacts quality of life by limiting swallowing, decreasing mobility and can cause death by choking. We conducted this study to quantify the burden of OPMD from the patient perspective. These data are needed to inform cost-effectiveness studies for treatments in development and to inform public policy.

**Methods:**

As part of the national Burden of Inherited and Inflammatory Neuromuscular Disease study survey, Canadians with OPMD were asked about the impact of OPMD on their health-related quality of life (Health Utilities Index Mark 3; HUI3), employment status, household finances, productivity (Work Productivity and Activity Impairment questionnaire), and out-of-pocket costs for assistive equipment and healthcare to help manage OPMD. Sociodemographic and epidemiological data were used to extrapolate participant responses into an estimate of the total patient-borne cost of OPMD for Canada.

**Results:**

Health-related quality of life was low in OPMD (*n* = 139, mean age 66 ± 10 years, 52% female) with HUI3 scores of 0.398 ± 0.323, compared to 0.826 for the normative population. Low HUI3 scores were associated with higher costs for assistive equipment and greater financial hardship. 29% of respondents with OPMD were in paid employment compared to 41% in the normative population. Impairments in total work productivity and non-work activity were 32 ± 27% and 61 ± 29%, respectively. Paid employment was associated with greater access to private insurance (odds ratio 4.2) which was in turn positively associated with the likelihood of visiting allied healthcare providers (odds ratio 2.5). The total patient-borne cost of OPMD for Canada was estimated to be $78.4 million (2026 Canadian dollars). Indirect costs accounted for 84% of the total; 53% for non-work activity impairment, 18% for loss of earning capacity, 12% for presenteeism and 2% for absenteeism. Direct costs comprised 16% of the total; 7% for healthcare visits and 9% for assistive equipment.

**Discussion:**

OPMD imposes substantial direct and indirect economic burden, primarily due to impact on work and productivity and less on equipment or health professional costs. The high indirect costs of OPMD should be a consideration in economic evaluations and future health policy decisions for emerging therapeutic interventions.

## 1 Introduction

Oculopharyngeal muscular dystrophy (OPMD; OMIM ID # 164300) is a genetic muscle disease characterized by dysphagia (swallowing difficulty), ptosis (drooping eyelids), and proximal muscle weakness, with age of symptom onset typically between 40 and 70 years old (1, 2). Most commonly, OPMD is inherited in an autosomal dominant pattern, though recessive cases have been identified (3–5). The underlying cause of OPMD is most commonly an expansion of the number of GCN repeats in the poly(A)-binding protein nuclear 1 (*PABPN1*) gene, where 10 repeats are typical and 11–18 repeats are associated with OPMD (3, 6). Rarely, OPMD can be caused by a point mutation that converts glycine at amino acid position 12 to alanine, resulting in a *de facto* expansion of the alanine sequence in PABPN1, linking the alanine residues found at positions 13 and 14 to those found at positions 2–11 (7, 8). While OPMD is rare globally (~1 in 100,000) (9), there are regions with far higher prevalence due to founder effects (1). The largest affected OPMD population is found among French Canadians, with an estimated 7,700 people carrying the *PABPN1* genetic expansion, based on prevalence of 1 in 1,000 (10), a population of approximately 7.7 million (11), and 100% penetrance by age 70 for the predominant (GCN)13 genotype (12). OPMD incidence is also elevated in the Rio Grande Basin of the United States (13), in the Canary Islands (14), among Bukhara Jews in Israel (15), and in Yakutia (16, 17).

While there is no cure or disease modifying therapy currently available, there are potential treatments in development (e.g., clintrials.gov NCT06185673). Disease management is currently based on multidisciplinary care: physical and functional evaluations (18–23), interventions for dysphagia (esophageal dilation, cricopharyngeal myotomy, percutaneous endoscopic gastrostomy) and levator/sling surgeries for ptosis), and medical equipment (walkers/wheelchairs) to support progressive mobility limitations (12, 24). While formal evaluations on the economic impact of OPMD have been limited, families living with OPMD face substantial costs including increased need for medical equipment, formal or informal caregiving, and reduced capacity to perform work and tasks of daily living. Understanding the socioeconomic burden of OPMD is essential to identify costs that impact health and quality of life (17, 18). Importantly, quantification the economic burden of OPMD and other neuromuscular diseases (NMD) is needed to inform cost effectiveness studies for treatments in development (18–23). Given that expensive treatments can be cost-effective if they reduce long-term costs borne by individuals and society, rigorous economic evaluations build the foundations for evidence-based healthcare policies and subsequent resource allocation to improve clinical outcomes and health-related quality of life (HRQoL). HRQoL data capture consequences that healthcare expenditures alone may miss, such as disability, reduced independence, caregiver burden, and limitations in work or education. While HRQoL is inherently a qualitative description of one’s overall health, quantitative HRQoL scales (e.g., health utility scores (25), quality adjusted life expectancy (26)) have been developed to help estimate cost-effectiveness and inform reimbursement or pricing decisions.

While economic evaluations are commonly conducted from the payer or societal level, patient perspectives are needed to determine if a treatment or intervention is actually affordable, feasible, adherable, or impactful (27, 28). With public spending under increasing scrutiny, and treatments for orphan diseases often carrying substantial costs (29, 30), accurate estimates of the indirect costs associated with genetic conditions are essential to inform health policy. While patient experiences and impairments associated with OPMD have been described previously (31–33), the patient-borne costs of OPMD have not been quantified. This study was conducted to assess the socioeconomic burden in OPMD from the patient perspective with the following specific aims: (1) evaluate health utility scores and QALE for OPMD in relation to population normative values, (2) relate health utility scores to indicators of financial wellbeing in OPMD, and (3) estimate the total direct and indirect costs faced by adults with OPMD in Canada.

## 2 Methods and materials

### 2.1 Overview

This study was part of the Burden of Inherited and Inflammatory Neuromuscular Disease (BIND) study, with detailed methodology described previously (34). BIND is a national, cross-sectional survey of individuals living with a NMD and their caregivers. The study protocol was approved by the Ottawa Health Science Network Research Ethics Board (Protocol ID # 20210601-01H). All participants had access to the informed consent form in both English and French, and provision of informed consent was implied by completion of the online survey. The survey was conducted either online or by telephone with assistance from trained study personnel at Muscular Dystrophy Canada (MDC). Those not capable of completing the survey themselves were allowed to have someone complete the survey with them on their behalf. 80% of participants had their diagnosis verified by MDC (31). Inclusion criteria for the current sub-study were that the respondent must be a resident of Canada, have reached age 18 years or over, be fluent in English and/or French and have self-reported with a diagnosis of OPMD. Participants were asked about sociodemographic, disease, and health utility characteristics, as well as their direct and indirect costs of living with OPMD. Except where stated otherwise, reported costs are in 2026 Canadian dollars, reflecting inflation-adjustment using a conversion factor of 1.0789 for the 2023 to 2026 time period (35).

### 2.2 Sociodemographic data

Participants were asked their age, age at diagnosis, sex at birth, highest level of education attained, employment status, household income, marital status, and if they had access to private insurance. Participants were also asked about their province/territory of residence and urban/rural designations based on partial postal code, known as ‘forward sorting area’ (36). Participants were also asked “to which ethnic or cultural groups did your ancestors belong?,” and this question was interpreted according to the racialized visible minority variable used in the Canadian census (37). Dropdown menus were used to collect information about education attained (10 levels collected, but condensed to binary for analysis; with *vs* without a Bachelor’s degree or higher) and household income (16 levels ranging from >$5,000 to ≥$250,000; 2023 Canadian dollars).

### 2.3 Health utility scores

To assess health status and health-related quality of life, participants completed the standardized and validated Health Utilities Index Mark 3 (HUI3) questionnaire (38). Single attribute scores (vision, hearing, speech, ambulation, dexterity, emotion, cognition, and pain/discomfort) range from 0–1.00, with 1.00 considered ideal. Multi-attribute scores range from −0.36 (worse than death) through 0 (equivalent to death), to 1.00 (perfect health) (25). HUI3 scores were compared to age- and sex-matched Canadian reference data (39), and used to estimate quality-adjusted life expectancy (QALE) in OPMD and the general public by multiplying HUI3 scores by remaining total life expectancy (26, 40). Remaining total life expectancy was obtained from sex- and age-matched Canadian normative data (41), noting that the (GCN)13 genotype has not been reported to impact life expectancy in OPMD (42).

### 2.4 Direct costs of OPMD

Direct costs of OPMD encompassed costs of healthcare visits as well as costs of medical equipment needed to help manage symptoms of OPMD. Participants were asked to report how many visits they had with various healthcare providers/services in the past 6 months. Costs associated with travel to attend visits with primary care/specialist physician were collected. Participants were asked if they incurred out-of-pocket costs for other healthcare visit types. Costs per visit were calculated based on fee recommendation by professional associations of healthcare providers, as described in detail previously (43). Participants listed specific costs spent on various types of medical equipment related to OPMD in the past 5 years.

### 2.5 Indirect costs of OPMD

The assessed indirect costs of OPMD included lost productivity at and away from work. As described previously (43), participants competed an adapted version of the Work Productivity and Activity Impairment questionnaire (WPAI) (44), measuring impairment while working (presenteeism), time missed from work (absenteeism), and activity impairment outside of work due to OPMD. Based on recommendations of our patient partners and previous work (45), the BIND survey also included productivity while at school, and questions on the type and quality of work performed and used a 6-month recall period. Additionally, we assessed loss of earning capacity among working aged individuals who reported complete inability to contribute to the labour force and earn wages due to OPMD. The monetary costs of presenteeism, absenteeism, and loss of earning capacity were calculated by indexing productivity losses to the median salary in 2023 for a person of the same age and sex (46). The monetary cost of non-work activity impairment was indexed to 35% of the median salary value, a procedure that has been used previously to monetize the time spent on caregiving activities (47), time spent commuting (48), and in our prior work in BIND (43).

### 2.6 Indicators of financial wellbeing

Participants were asked to complete the Comprehensive Score for Financial Toxicity (COST) – Functional Assessment of Chronic Illness Therapy (FACIT) questionnaire (49). The COST-FACIT questionnaire consists of 11 questions rated on a 5-point Likert scale (0 = not at all, 4 = very much), which are summed to obtain a COST score ranging from 0 to 44. Lower scores indicate worse financial toxicity. A 12th question serves as a parallel measure to the COST score and asks respondents to rate the hardship imposed by their condition on their family. COST-FACIT has demonstrated strong reliability and validity when assessing financial toxicity in conditions such as cancer (50), diabetes (51), and chronic disease (52). We have previously reported COST-FACIT scores and assessed their determinants in the broader BIND cohort (53), but have not examined heterogeneity existing within specific NMDs. Interpretations of COST-FACIT scores were as follows: ≥26 indicated no financial toxicity, 14–25 indicated mild financial toxicity, 1–13 indicated moderate financial toxicity, and 0 indicated severe financial toxicity (54). Participants were also asked a series of “yes/no” questions about interactions between their financial situation and management of their NMD.

### 2.7 Population-based cost estimates

Annual direct and indirect costs of OPMD in Canada were estimated by multiplying the per-person cost by the estimated number of individuals with OPMD expected to incur each type of cost. The cost estimates were generated using a series of evidence-based assumptions. As French Canadians account for the vast majority of people with OPMD in Canada, we began with a total population of 7,728,465 individuals with French as the first official language spoken (11). By applying a prevalence rate of 1 in 1,000 (10) with 100% penetrance for the (GCN)13 genotype by age 70 (12), an estimated 7,728 living Canadians will be affected by OPMD in their lifetime. However, because OPMD is a late-onset condition, younger people with OPMD do not experience any symptoms. While individual experiences with OPMD will vary considerably, mean ages for onset of ptosis and dysphagia have been reported as 48 years and 50 years, respectively (12). For population-based cost estimates, we used a more conservative estimate that the symptoms of autosomal dominant OPMD are not burdensome until age 55 years on average. Among those with French as the first official language 36.3% are age 55 years and older, 15.6% are working age (55–64 years) and 20.6% have reached Canada’s age of retirement (65 years) (11). These data suggest that 2,802 individuals in Canada are burdened by symptoms of OPMD, of whom 1,206 can be considered working age, and 1,596 can be considered to have reached the age of retirement.

Healthcare and equipment costs were assumed to apply to all those burdened by OPMD (2,802 individuals). We assumed that the employment status of our sample of people with OPMD age 55 to <65 years was representative of employment status in the broader population with OPMD in this age range, and the remaining 1,206 individuals were subdivided accordingly. The specific employment status categories used, and associated costs applied, were: age ≥65 years (non-work activity impairment), working for pay (presenteeism, absenteeism, non-work activity impairment), studying (non-work activity impairment), neither working nor studying due to OPMD (loss of earning capacity, non-work activity impairment), and neither working nor studying for reasons other than OPMD (non-work activity impairment).

### 2.8 Statistical comparisons

HUI3 scores were compared between participants who responded “yes” *vs* “no” to the questions probing interactions between NMD care and financial situation using Student’s *t*-tests (2-tailed, independent samples). Relationships between COST-FACIT scores and HUI3 scores were assessed using linear regression (Pearson’s *r*). The relationship between household income and HUI3 scores was assessed using linear regression (Spearman’s *ρ*). HUI3 scores were compared between different categorical levels of financial toxicity and different levels of financial hardship imposed by OPMD using one-way ANOVAs followed by Tukey’s post-hoc testing to compare specific means. Relationships of age and HUI3 with direct and indirect costs were assessed using non-parametric correlation analysis (Spearman’s *ρ*). Sex differences in direct and indirect costs were probed using Mann–Whitney U tests. The relationships of private health insurance with allied healthcare visits and employment status were explored using Fisher’s exact test for retrospective data, and results are reported as an odds ratio. Statistical significance was accepted at a level of *α* = 0.05. Analyses were conducted using complete-case analysis, whereby observations with missing data for variables required for a given analysis were excluded from that analysis.

## 3 Results

### 3.1 Participant characteristics, with reference to population norms

Surveys were received from 139 individuals with OPMD [52% female, *n* = 3 no response, (NR)], of whom, 17 indicated that they were also caregivers for someone else with a NMD. Mean ± standard deviation (SD) age of respondents was 66 ± 10 years. This mean age was the second highest for all NMDs in the BIND study (behind only inclusion body myositis), the reported age data conform to expectations for OPMD as a late-onset NMD. Mean±SD age at diagnosis was 51 ± 8 years, corresponding to a mean±SD time since diagnosis of 14 ± 10 years for the cohort. Consistent with the association of OPMD with French-Canadian ancestry, 77% of surveys were completed in French, 81% of respondents spoke French at home (92/113), and 81% (112/139) resided in the province of Quebec. An additional 4% (5/139) resided in districts of Eastern Ontario with substantial populations (>20%) listing French as their mother tongue (55). Seven responses indicated both Indigenous and French-Canadian ancestry and 106 responses indicated White/Caucasian ancestry, no responses indicated racialized visible minority, and 26 surveys had no response for this question. The proportion of respondents living in a rural area (7%) was considerably lower than that of the general populations of Canada (16%) and Quebec (18%) (56). The proportion of respondents in paid employment was 29%, compared to 41% for the age- and sex-matched general population (57). In 2023 Canadian dollars, the median [interquartile range; IQR] household income of respondents was $55,000 [$35,000–$85,000], lower than that of the general population ($83,600 for Canada, $77,500 for Quebec), including that of households with the major income earner being greater than 65 years old ($84,700 for Canada, $75,500 for Quebec) (58). 44% of respondents held a degree at Bachelor’s level or higher, greater than the 24% found among Canadians age 45 years and older (59). The 68% proportion of respondents who were married or in a common-law relationship was comparable to the 67% rate found in the age-matched general public (60) (Table 1).

**Table 1:** Demographic and socioeconomic characteristics of participants living with oculopharyngeal muscular dystrophy (OPMD).

Demographics	Value
Sex at birth (*n* = 136, 3 NR)	*n* = 72 Female, *n* = 64 Male
Age at diagnosis (*n* = 119, 20 NR), mean±SD	51 ± 8 years
Current age (*n* = 134; 5 NR), years, mean±SD	66 ± 10 years
Current age ≥65 years	*n* = 74 (55%)
Time since diagnosis (*n* = 116; 23 NR), years, mean±SD	14.1 ± 9.8 years
Ancestry (*n* = 113; 26 NR)	
White/Caucasian, including French Canadian	*n* = 106 (94%)
Both Indigenous and French-Canadian	*n* = 7 (6%)
Marital status (*n* = 116; 23 NR): % married or common-law	68%
Education level (*n* = 113, NR = 26)	
Below bachelor degree	*n* = 67 (59%)
Bachelor degree and above	*n* = 46 (41%)
Annual household income (in 2023 Canadian dollars; *n* = 95; 44 NR)	
< $20,000	*n* = 12 (13%)
$20,000–$50,000$	*n* = 27 (28%)
$50,000–$100,000	*n* = 37 (39%)
$100,000–$150,00	*n* = 8 (8%)
>$150,000	*n* = 11 (12%)

SD, standard deviation; NR, no response.

### 3.2 Health utilities and quality-adjusted life expectancy

The HUI3 multi-attribute score indicated low HRQoL for OPMD, with mean±SD of 0.398 ± 0.323 (Figure 1A), substantially lower than the mean score of 0.826 for of the age-and sex-matched general population (39). In subset analysis, significant differences in multi-attribute HUI3 scores were identified between individuals aged 62- < 68 years and individuals aged ≥74 years (*p* = 0.04) as expected given progressive nature of OPMD with worsening dysphagia, ptosis, and weakness as well as general aging. The mean±SD difference in HUI3 multi-attribute score between OPMD and the age- and sex-matched general population ranged from 0.139 ± 0.285 among females age 62 to <68 years to 0.585 ± 0.153 among males age ≥74 years (Table 2). Corresponding reductions in QALE attributable to OPMD ranged from 13.6 ± 10.2 years among females age ≤62 years to 2.9 ± 5.8 years among females age 62 to <68 years (Table 2). Contrary to expectations for a progressive condition, age had a negligible relationship with the difference between HUI3 score in OPMD and HUI3 score from the age and sex-matched general population (Pearson’s *r* = −0.089; Figure 1B). Notable though, was the finding that HUI3 scores below 0 (considered worse than death) were predominantly found among younger individuals; 5 of the 10 were less than 62 years of age, and the remaining 5 were 63, 67, 70, 85, and 86 years old.

**Figure 1:**
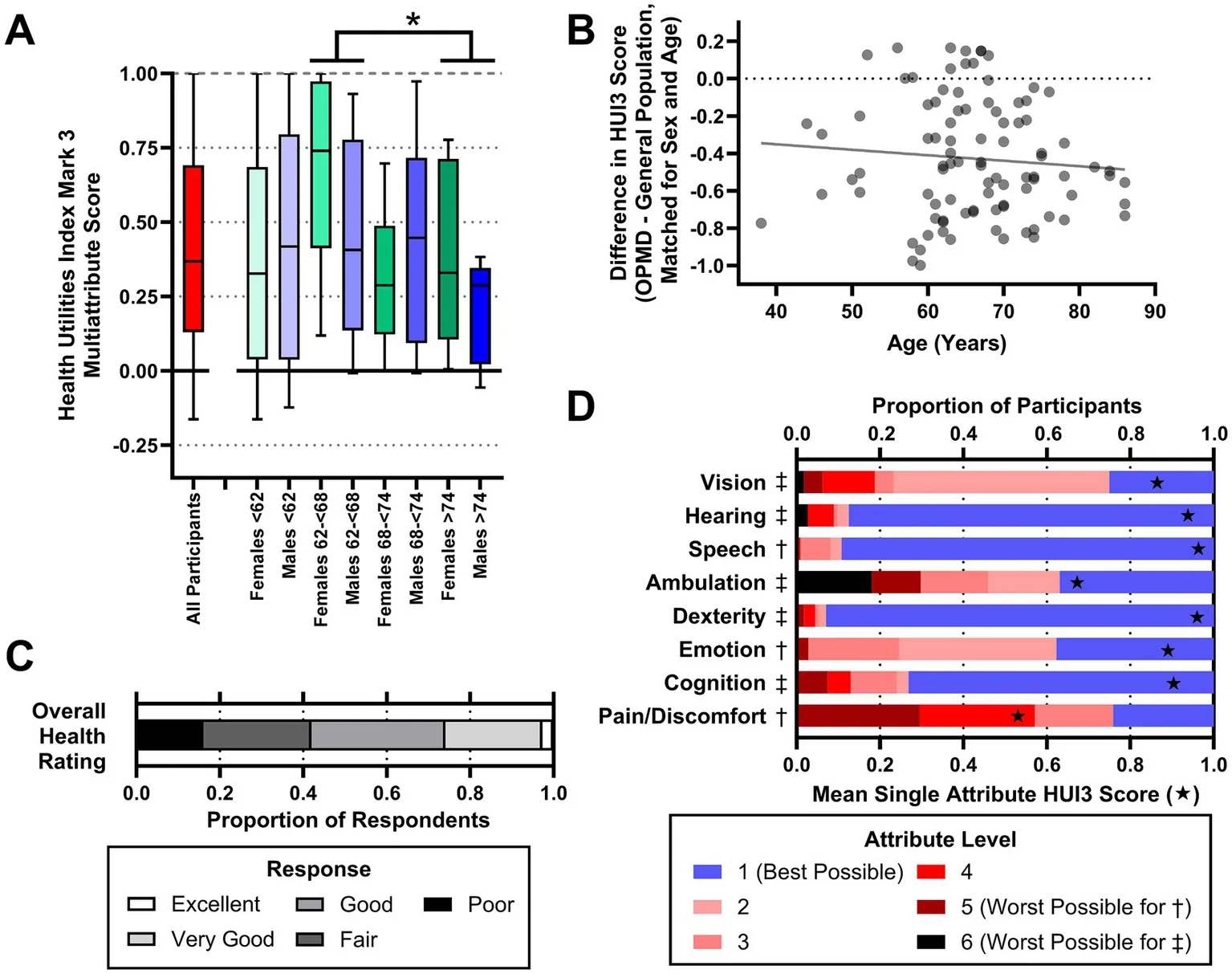
Health Utilities Index Mark 3 (HUI3) scores for participants with oculopharyngeal muscular dystrophy (OPMD). **(A)** Box and whisker plots showing HUI3 multi-attribute score for all participants and stratified by age and sex. The levels multi-attribute scores were compared between subgroups using a 2-way analysis of variance (ANOVA), with a main effect of age identified (*p* = 0.04). Those 62- < 68 years of age were found to be significantly different than those with age ≥74 years (*p* = 0.04) in post-hoc testing (Tukey’s HSD); marked with an asterisk. Neither sex (*p* = 0.45) nor the interaction between sex and age (*p* = 0.19) were statistically significant. **(B)** Scatterplot depicting the relationship between age and the difference between HUI3 scores among participants with OPMD and HUI3 scores in the general population (age- and sex-matched at the individual level). Linear fit is shown (Pearson’s *r* = 0.079). **(C)** Distribution of participants’ ratings of their overall health. **(D)** Mean (★) single attribute HUI3 score for all participants, and proportional distribution of participants across levels of each attribute (colored bars).

**Table 2:** Health utility scores and quality-adjusted life expectancy in OPMD in comparison to Canadian population norms; (mean ± standard deviation).

Age bracket ^a^ (Years)	*n*	Age-adjusted life expectancy ^b^ (Years)	HUI3 Multi-attribute score for OPMD	HUI3 Multi-attribute score for Normative Population ^c^	QALE for OPMD ^d^ (Years)	QALE for normative population ^d^ (Years)	OPMD-associated decline in QALE (Years)
Males							
[≤62 years] 56.1 ± 7.1	6	27.5 ± 7.5	0.422 ± 0.435	0.863	12.9 ± 14.1	23.9 ± 7.2	−10.9 ± 10.3
[63–67 years] 64.5 ± 2.0	16	19.9 ± 1.5	0.414 ± 0.289	0.851	8.2 ± 5.8	16.9 ± 1.3	−8.7 ± 5.8
[68–73 years] 70.5 ± 1.7	10	15.3 ± 1.3	0.434 ± 0.3298	0.849	6.7 ± 5.3	13.0 ± 1.1	−6.3 ± 5.2
[≥74 years] 79.9 ± 5.4	10	9.3 ± 3.1	0.160 ± 0.192	0.745	1.7 ± 2.1	7.2 ± 3.0	−5.4 ± 2.5
Females							
[≤62 years] 55.6 ± 6.4	21	28.2 ± 5.8	0.365 ± 0.346	0.842	10.2 ± 9.3	23.8 ± 5.2	−13.6 ± 10.2
[63–67 years] 65.2 ± 2.1	13	19.9 ± 1.9	0.690 ± 0.284	0.829	13.6 ± 5.4	16.5 ± 1.7	−2.9 ± 5.8
[68–73 years] 71.3 ± 2.1	11	15.3 ± 2.1	0.293 ± 0.226	0.825	4.5 ± 3.3	12.6 ± 1.7	−8.1 ± 3.3
[≥74 years] 78.1 ± 3.0	7	10.7 ± 2.1	0.359 ± 0.285	0.771	4.0 ± 3.6	8.3 ± 1.7	−4.3 ± 3.1

^a^Age brackets were defined to include at least 6 HUI3 multi-attribute scores per category.

^b^Calculated based on life expectancy at age data from Statistic Canada, controlling for sex and province of residence. The Canadian mean was used when province or territory-specific data were not available (34). The stated value applies to both OPMD and the normative (i.e., sex-matched and age-matched) Canadian population.

^c^Data are matched for the age and sex of the participants with OPMD (39).

^d^QALE = HUI3 score age-adjusted life expectancy.

Only 3 of 112 respondents rated their overall health as excellent, while 26 rated it as very good, 36 as good, 29 as fair, and 18 as poor (Figure 1C). The lowest (i.e., worst) single-attribute HUI3 scores were for pain/discomfort (0.51831 ± 0.386) and ambulation (0.639 ± 0.395), with all other single-attribute scores above 0.850 on average (Figure 1D). Single attribute HUI3 scores stratified by age and sex are shown in Supplementary Figure S1.

### 3.3 Indicators of financial toxicity and relationship with health utility scores

Over half of respondents (54%) did not have private insurance (contrast with 18% in the general population of Quebec, 30% in the subset at age 65 years (61)). In addition, one third (33%) had to take money out of retirement or savings for NMD care, 11% have relocated to be closer to NMD care or support, 12% missed a medication or treatment for financial constraints, 8% missed a clinical visit for financial reasons, and 38% received a government disability tax credit (Table 3). Answers suggestive of financial stress in OPMD were associated with lower HUI3 scores, reaching statistical significance among respondents that have taken money out of savings or retirement (HUI3 of 0.144 *vs* 0.527), missed a medication or treatment for financial reasons (HUI3 of 0.196 *vs* 0.418), or received a disability tax credit (HUI3 of 0.253 *vs* 0.481) (Table 3). Greater financial toxicity (i.e., lower COST-FACIT scores) was associated with lower HUI3 scores, with Pearson’s *r* of 0.536 (*p* < 0.0001). Similarly, HUI3 scores were significantly (*p* < 0.01) lower among those with moderate financial toxicity (0.087 ± 0.180) than those with mild (0.390 ± 0.291) or no financial toxicity (0.523 ± 0.318) (Table 4). Those who responded “not at all” to the statement “my NMD has been a financial hardship to my family and me” had mean±SD HUI3 scores of 0.562 ± 0.295, significantly (*p* < 0.05) better than the HUI3 scores associated with any other response (Table 4). Better HRQoL (i.e., higher HUI3) was associated with higher household income (Spearman’s *ρ* = 0.36; *p* < 0.001).

**Table 3:** Indicators of financial wellbeing in relation to health utility scores in OPMD.

Query		Do you have private insurance?	Have you taken money from savings or retirement funds for OPMD care/support?	Have you relocated to be closer to NMD care or support?	Have you ever skipped a medication or medical treatment because you could not afford it?	Have you ever skipped a clinical visit or doctor’s appointment because you could not afford to attend?	Have you visited with a healthcare provider in the past 6 months (excluding primary care or specialist physician)?	Do you receive disability tax credit?
Responded yes to query	Percentage of Respondents	46%	33%	11%	12%	8%	72%	38%
	*n* Yes/Total *n*	54/118	38/115	13/115	14/115	9/114	64/89	44/115
	HUI3 Score (mean±SD)	0.447 ± 0.318	0.144 ± 0.198	0.229 ± 0.333	0.196 ± 0.296	0.310 ± 0.366	0.407 ± 0.334	0.253 ± 0.310
Responded no to query	Percentage of Respondents	54%	67%	89%	88%	92%	28%	62%
	*n* No/Total *n*	64/118	77/115	102/115	101/115	105/114	25/89	71/115
	HUI3 Score (mean±SD)	0.351 ± 0.323	0.527 ± 0.298	0.410 ± 0.320	0.418 ± 0.32	0.401 ± 0.322	0.382 ± 0.313	0.481 ± 0.302
Comparison of HUI3 Scores (Yes vs No)	*p*-value in *t-*test	0.14	<0.0001	0.13	0.039	0.51	0.69	<0.001

**Table 4:** HUI3 scores in relation to financial hardship and financial toxicity in OPMD.

Measure	HUI3 score Mean ± SD	*n*
OPMD has been a financial hardship to my family and me		
Not at all	0.562 ± 0.295	54
A little bit	0.272 ± 0.216*	12
Somewhat	0.185 ± 0.283*	20
Quite a bit	0.120 ± 0.113*	4
Very much	0.138 ± 0.133*	8
Level of financial toxicity		
None (COST-FACIT Score 26–44)	0.523 ± 0.318	43
Mild (COST-FACIT Score 14–25)	0.390 ± 0.291	38
Moderate (COST-FACIT Score 1–13)	0.087 ± 0.180†‡	17

*p < 0.05 vs. Not at all.

† − *p* < 0.01 vs. Mild financial toxicity.

‡ − *p* < 0.001 vs. No financial toxicity.

### 3.4 Costs of living with OPMD

#### 3.4.1 Direct costs of medical equipment

Over half of respondents (51/88; 58%) reported out-of-pocket costs for medical equipment for OPMD management in the past 5 years (Table 5). Those with out-of-pocket costs were significantly (*p* < 0.001) older (69.5 ± 9.6 years; 52% female) than those without out-of-pocket costs (60.0 ± 7.8 years; 64% female). Total equipment costs did not differ significantly between males and females (*p* = 0.53). Among respondents incurring out-of-pocket costs for medical equipment, the median annualized expenditure was $895 (IQR, $151–$2,104). When all respondents were included, regardless of out-of-pocket spending, the median annualized cost decreased to $73 (IQR, $0–$1,223) (Table 5). The most common equipment costs incurred were home modifications (26%), orthopedic equipment (25%), and new power wheelchairs (24%) (Table 5). Equipment categories with the highest out-of-pocket costs were vehicle modifications (annualized cost of $6,236 [$129–$14,619]; incurred by 3/88 respondents), home modifications (annualized cost of $1,726 [$432–$3,237]), and new power wheelchairs (annualized cost of $841 [$432–$1,079]) (Table 5). There was a significant negative association between equipment costs and HUI3 score (Spearman’s *ρ* = −0.50, *p* < 0.0001), and a significant positive association between equipment costs and age (Spearman’s *ρ* = 0.50, *p* < 0.0001).

**Table 5:** Equipment costs in participants living with oculopharyngeal muscular dystrophy (OPMD).

Type of equipment or device	Participants with incurred cost, 5-year recall (%)	Annualized out-of-pocket costs (Median [IQR])
Home rehabilitation/renovation	26 (*n* = 23/88)	$1,726 [$432–$3,237]
Hospital bed/mattress	14 (*n* = 12/88)	$432 [$205–$812]
Other assistive devices/ medical equipment	15 (*n* = 13/88)	$324 [$43–$1,942]
New manual wheelchair	11 (*n* = 10/88)	$102 [$65–$221]
Repair of manual wheelchair	1 (*n* = 1/88)	$108 [$0–$0]
New power wheelchair/scooter	24 (*n* = 21/88)	$842 [$432–$1,079]
Repair of power wheelchair/scooter	15 (*n* = 13/88)	$216 [$108–$259]
Orthopedic equipment (e.g., leg braces, ankle-foot orthotics, walkers etc.)	25 (*n* = 22/88)	$92 [$46–$127]
Non-invasive ventilation devices (e.g., CPAP, BiPAP, CoughAssist etc.)	3 (*n* = 3/88)	$356 [$189–$394]
Vehicle modifications	3 (*n* = 3/88)	$6,236 [$129–$14,619]
Total equipment cost per respondent incurring a cost	58 (*n* = 51/88)	$895 [$151–$2,104]
Total equipment cost per respondent		$73 [$0–$1,223]

Costs are reported in 2026 Canadian dollars.

#### 3.4.2 Direct costs of healthcare visits

Visits with primary care/specialist physicians/nurse practitioners are publicly funded in Canada, and 46% (*n* = 53/116) of respondents reported visiting with this type of healthcare provider in the preceding 6 months, with median [IQR] of 2 [1–3] visits per person. Costs associated with travel to these appointments amounted to $54 ± $135 across all participants, with median [IQR] of $22 [$0 – $29], noting that a small number of participants reported high travel costs and stated the need for overnight accommodations and/or transportation by ferry to attend visits.

Usage and costs associated with visits with healthcare providers that are typically not publicly covered (allied healthcare providers) are shown in Table 6. Seventy-two percent (*n* = 64/89) of participants indicated that they visited with at least one allied healthcare provider in the past 6 months to help manage their OPMD. The most common types of visits were with physio/occupational therapists (33%; *n* = 29/89), massage therapists (20%; *n* = 18/89), dieticians/nutritionists (17%; *n* = 15/89), and chiropractors/osteopaths (17%; *n* = 15/89). The most frequently visited allied healthcare providers in the preceding 6 months were psychologists/psychiatrists/psychotherapists with 8 [4-11] visits per user, personal care attendants/personal support workers with 6 [2-10] visits per user, and chiropractor/osteopath with 6 [2-6] visits per user (all values are median [IQR]). All other types of allied healthcare providers were visited 3 times or fewer (median) in the preceding 6 months. Sex (*p* = 0.067), age (Spearman’s *ρ* = 0.094, *p* = 0.39), and HUI3 score (Spearman’s *ρ* = −0.144, *p* = 0.22) were not significantly associated with the total number of allied healthcare provider visits in the past 6 months. Access to private insurance increased the likelihood of visiting with any allied healthcare provider in the past 6 months with an odds ratio of 2.45 (*p* < 0.05), and having private insurance was associated with employment, with an odds ratio of 4.18 (*p* < 0.001).

**Table 6:** Allied healthcare visits and associated costs in participants living with oculopharyngeal muscular dystrophy (OPMD).

Allied healthcare provider type	Reported visits and cost occurrence				Predicted visit costs	
	Participants with utilization in the past 6 months	Visits per user in past 6 months median [IQR]	Users with OOP Cost	Visits with OOP Cost	Cost per visit (43)	Annualized Cost for All Users Median [IQR]
Personal care attendant/Personal support worker	6% *n* = 5/89; 50 NR	6 [2–10]	50% *n* = 2/4; 1 NR	91% *n* = 70/77; 2 NR	$120	$1,440 [$480–$2,400]
Massage therapy	20% *n* = 18/89; 50 NR	3 [2–6]	94% *n* = 17/18; 0 NR	93% *n* = 76/82; 0 NR	$112	$672 [$448–$1,344]
Physiotherapist/Occupational therapist	33% *n* = 29/89; 50 NR	2 [2–6]	48% *n* = 12/25; 4 NR	61% *n* = 79/130; 17 NR	$152	$608 [$608–$1,824]
Chiropractor/Osteopath	17% *n* = 15/89; 50 NR	6 [2–6]	93% *n* = 13/14; 1 NR	99% *n* = 90/9; 1 NR	$254	$3,048 [$1,016–$3,048]
Psychologist/Psychiatrist/Psychotherapist	9% *n* = 8/89; 50 NR	8 [4–11]	57% *n* = 4/7; 1 NR	54% *n* = 33/61; 10 NR	$250	$4,000 [$2,000–$5,250]
Dietitian/Nutritionist	17% *n* = 15/89; 50 NR	1 [1–4]	42% *n* = 5/12; 3 NR	30% *n* = 10/33; 4 NR	$80	$160 [$160–$560]
Respiratory therapist	3% *n* = 3/89; 50 NR	1 [1–1]	50% *n* = 1/2; 1 NR	50% *n* = 1/2; 1 NR	$50	$100 [$100–$100]
Naturopathy/Alternative therapies	6% *n* = 5/89; 50 NR	2 [2–2]	100% *n* = 5/5; 0 NR	100% *n* = 17/17; 0 NR	$200	$800 [$800–$800]
Combined total	72% *n* = 64/89; 50 NR	5 [2–10]	72% *n* = 42/58; 6 NR	76% *n* = 376/493; 35 NR	NA	Users Only (*n* = 64) $1,344 [$668–$3,482]
						Users and Non-Users (*n* = 89) $608 [$0–$2,688]

Costs are reported in 2026 Canadian dollars.

IQR, interquartile range; NR, no response; N/A, not applicable; OOP, out of pocket.

Out-of-pocket costs were reported by over 90% of individuals visiting massage therapists, chiropractors/osteopaths, and naturopaths/providers of alternative therapies. For all other allied healthcare providers (e.g., physiotherapists), 40 to 60% of participants indicated out-of-pocket cost associated with their visit(s). Using cost of visit values provided by professional organizations, as described previously (43) and the visit frequencies reported by participants, the allied healthcare providers with the highest annual cost per median use were psychologist/psychiatrist/psychotherapist ($4,000) chiropractor/osteopath ($3,048), and personal care attendant/personal support worker ($1,440). All allied healthcare providers had an annual cost below $1,000 based on median use. The annual per-person median [IQR] cost of all allied healthcare provider visits was $1,344 [$668–$3,482] among those with at least one visit (*n* = 64; median [IQR] of 5 [2–10] visits with any allied healthcare provider), or $608 [$0–$2,688] when including those with no visits with allied healthcare providers (*n* = 25 for a total *n* = 89).

#### 3.4.3 Indirect costs

Results of the WPAI questionnaire are summarized in Table 7. Absenteeism due to OPMD was reported by 40% of respondents in paid employment (*n* = 10/25), amounting to 4 ± 8% of total work time. Presenteeism was reported by 84% of participants in paid employment (*n* = 21/25), corresponding to a 29 ± 27% reduction in productivity at work. Total work impairment was 32 ± 27%. Eight of the 60 respondents under 65 years of age indicated that they were not working at all due to OPMD; these 8 participants were considered to have a 100% loss of earning capacity. Impairment in non-work activity was 55 ± 26% in those working for pay, 61 ± 29% among those studying, 54 ± 33% among those under age 65 years who were neither working nor studying, and 68 ± 27% among those aged 65 years and older.

**Table 7:** Summary of individual and population-level cost estimates for individuals living with oculopharyngeal muscular dystrophy (OPMD) in Canada.

Cost type	Participant subset		Specific cost category	BIND survey data			Annualized cost estimate per respondent in subset		Extrapolation to Canadian OPMD population		
				Number of respondents	Cost Prevalence % (*n*)	Percent impairment (Mean ± SD)	Mean ± SD	Median [IQR]	Estimated number of Canadians with this cost ^a^	Estimated total annual cost in Canada ^a^	Estimated Cost Subtotals and Totals for Canada
Indirect Costs	In paid employment		Overall Work Impairment (Absenteeism + Presenteeism)	25 (6 NR)	84 (*n* = 21/25)	32 ± 27	$19,509 ± $18,350	$13,465 [$5,809–$27,050]	554	$10,807,000	$17,413,000
			Absenteeism	25 (6 NR)	40 (*n* = 10/25)	4 ± 8	$2,361 ± $4,400	$0 [$0–$3,189]	554	$1,308,000 (Reflected in Overall Work Impairment)	
			Presenteeism	25 (6 NR)	84 (*n* = 21/25)	29 ± 27	$17,148 ± $16,696	$12,118 [$5–$23,722]	554	$9,500,000 (Reflected in Overall Work Impairment)	
			Non-work activity impairment	31 (0 NR)	94 (*n* = 29/31)	55 ± 26	$11,924 ± $5,800	$12,439 [$7,108–$16,494]	554	$6,606,000	
	Not in paid employment	Studying	Non-work activity impairment	11 (0 NR)	91 (*n* = 10/11)	61 ± 29	$12,233 ± $5,937	$12,439 [$10,662–$14,197]	163	$1,994,000	$48,508,000
		Age 18–64 years; Not studying	Loss of Earning Capacity	18 (0 NR)	44 (*n* = 8/18)	100 ^b^	$52,842 ± $5,852 ^b^	$50,773 [$50,773–$50,773] ^b^	261	$13,792,000	
			Non-work activity impairment	18 (0 NR)	89 (*n* = 16/18)	54 ± 33	$20,209 ± $3,233	$17,771 [$17,771–$23,563]	489	$9,882,000	
		Age ≥65 years; Not Studying	Non-work activity impairment	51 (0 NR)	100 (*n* = 51/51)	68 ± 27	$14,312 ± $6,510	$14,138 [$9,139–$20,029]	1,596	$22,841,000	
Direct Costs	All participants		Allied healthcare visit costs	89 (50 NR)	72% (*n* = 64/89)	N/A	$1,992 ± $3,282	$608 [$0–$2,688]	2,802	$5,581,000	$12,489,000
			Assistive equipment costs	88 (26 NR; 25 not asked)	58% (*n* = 51/88)	N/A	$2,465 ± $9,981	$73 [$0–$1,223]	2,802	$6,908,000	
											Estimated Grand Total: $78,410,000

Costs are in 2026 Canadian dollars.

IQR, Interquartile range; N/A, not applicable; NR, no response; SD, standard deviation.

^a^Mean age at onset of symptom burden assumed to be 55 years for population-based estimates.

^b^Stated impairment and cost estimates at the individual level only reflect impacted individuals.

HUI3 score had significant negative associations with non-work activity impairment and total work impairment, with Spearman’s *ρ* values of −0.540 (*p* < 0.001) and −0.541 (*p* < 0.01), respectively. Sex was not significantly associated with impairment in non-work activity (*p* = 0.35) or total work impairment (*p* = 0.27). Age was not significantly associated with total work impairment (Spearman’s *ρ =* 0.13, *p* = 0.52) but was significantly and positively correlated with non-work activity impairment (Spearman’s *ρ =* 0.33, *p* < 0.001).

Indirect costs were assigned monetary value by indexing the percent impairments reported above to the median annual wage for Canadians of the same sex and age. Note that the cost estimates listed below apply only to the specific subsets of respondents anticipated to incur that cost type (see Table 7). Absenteeism had an annual cost of $2,361 ± $4,400 per person and presenteeism had an annual cost of $17,148 ± $16,696 per person, with total work impairment amounting to $19,509 ± $18,350 per person per year. Loss of earning capacity had a cost of $52,842 ± $5,852 per person per year. The monetary value of non-work impairment was discounted to a maximum possible value of 35% of the median wage for Canadians of the same sex and age (versus 100% of the median wage for productivity losses and loss of earning capacity). By this method, impairment in non-work activity had an annual cost per person of $11,924 ± $5,800 amongst those in paid employment, $12,233 ± $5,937 amongst those studying, $20,209 ± $3,233 amongst those aged 18–64 years and not working, and $14,312 ± $6,510 amongst those age 65 years and older. The monetary values listed above were used to estimate population-based cost estimates for OPMD in Canada.

### 3.5 Population-based cost estimate for living with OPMD

The grand total of all costs borne by the Canadian population with OPMD was estimated to be $78.4 million per year. In estimating national level costs of OPMD, we had to consider that OPMD is a late-onset condition and the cost of living with OPMD will vary with disease progression. Thus, our population-level cost estimates considered those with OPMD who are under 55 years of age to have no disease burden (noting that *individual experiences* with OPMD will deviate considerably in both positive and negative directions). All individuals with OPMD over age 55 years (estimated to be 2,802 Canadians) were considered to have burden associated with medical equipment ($6.9 million per year), healthcare visits ($5.6 million per year), and impairment outside of work (see below) (Table 7). Those between 55 and 64 years of age were considered to have an additional burden of loss of earning capacity if they were not working due to OPMD, or impairment while at work if they were in paid employment. To calculate these two additional costs, it was necessary to assume that the work status of our survey respondents aged 55–64 years was representative of the work status of the Canadian population with OPMD age 55–64 years (estimated to be 1,206 individuals). Among the 37 survey respondents age 55–64 years, 17 were working (translating to 554 people), 5 were studying (translating to 163 people), 8 were not working due to OPMD (translating to 261 people), and 7 were not working for reasons outside of OPMD (translating to 228 people). By the assumption of a representative sample, the overall cost of work impairment was estimated to be $10.8 million per year ($9.5 million from presenteeism and $1.3 million from absenteeism, with 554 Canadians impacted), and the cost associated with a complete loss in earning capacity due to OPMD was estimated to be $13.8 million (261 Canadians impacted) (Table 7). Impairment in activities outside of work also incorporated variability based on employment status and was estimated to be $41.3 million per year in total, with 2,802 Canadians impacted (Table 7). Consequences of choosing a different age cutoff (i.e., mean age at onset of symptom burden) on national cost estimates are shown in Supplementary Figure S2.

## 4 Discussion

This nationwide survey quantified the detrimental impact of OPMD on health utility scores and quality-adjusted life expectancy, establishes association between reduced health utility scores and financial hardship, and provides a population-level estimate for the costs imparted by OPMD on people living with this condition in Canada. We estimate that there are over 2,800 people living with symptomatic OPMD in Canada facing a total annual cost of $78.4 million in 2026 Canadian dollars. These estimates do not include the additional 4,900 living Canadians with the genetic variant that will cause them to develop OPMD symptoms and disability in their lifetime. The costs faced by people with symptomatic OPMD were impairment in non-work activity ($41.3 million), loss of earning capacity ($13.8 million), impairment in work activity ($10.8 million), healthcare visit costs ($5.6 million), and healthcare equipment costs ($6.9 million). On average, participants with OPMD had health utility scores (0.398 ± 0.323) that were substantially lower than the age- and sex-matched general public (0.826). Those experiencing financial hardship and/or financial toxicity due to OPMD and financial toxicity exhibited lower HRQoL on average, as indicated by HUI3 scores.

The primary single-attribute drivers of low HUI3 scores were ambulation and pain/discomfort. Ambulation is expected to be affected in OPMD, given lower > upper extremity proximal weakness that increases with disease progression and age (1, 2). Regarding pain/discomfort, 85 out of 112 (76%) respondents reported pain at level 3, 4, or 5 on the HUI3 scale, markedly higher than the 54% of respondents reporting pain in a Dutch OPMD cohort using McGill’s Pain Questionnaire (31). Neither the current study nor the Dutch study found age-dependence of pain prevalence, and the Dutch authors considered the 54% reporting pain to be remarkably high for OPMD. Differences between studies is unlikely to be attributed to genotype as the Dutch cohort was predominantly comprised of individuals with GCN 16 in *PABPN1* which has a more severe disease course than GCN 13 in *PABPN1* which predominates in Canada. The specific reason for differences in pain reported between studies is unknown, but it could lie in the specific wording of questions, with pain and discomfort considered equal and inseparable in the HUI3. As the earliest and most troubling symptoms of OPMD (dysphagia, ptosis, fatigue) are uncaptured by other components of the HUI3 questionnaire, it would not be unreasonable for a respondent to categorize the impact of these symptoms under “discomfort.” Alternatively, the pain/discomfort scores may include a “response shift” that occurs once patients facing a chronic disease have adapted to their new situation and changed their internal conceptualization of quality of life (62). Several studies on progressive adult-onset muscular dystrophies have reported comparable findings. For example, advancing age was associated with better mental composite scores on the SF-36 questionnaire in participants with OPMD (63), lower quality of life was reported among those with adult onset compared to juvenile onset myotonic dystrophy (64) and there was a lack of association between disease duration and quality of life in limb girdle muscular dystrophy (65). The early years of symptoms may also represent a major transitional period that diminishes the range of work and leisure activities one is able to perform, which could also impact household finances and psychological well-being. While psychological distress has been found to be comparable between adults with long-standing OPMD diagnosis, and the general population (31), there is well-established need for psychosocial interventions soon after diagnosis with a chronic disease (66), and assistance navigating the early years of OPMD symptoms could be an unmet need. For example, Canada’s National Population Health Survey was used to study the impact of depression on HUI3 scores in Canada, and HUI3 scores were found to be reduced by approximately 0.22 (averaged across ages), and clinically meaningful reductions in emotion, cognition, and pain subscores were identified (67). The possible benefit of allocating resources to help patients cope with their diagnosis, associated lifestyle changes, and navigating workplace accommodations should be investigated in future work. Finally, our OPMD cohort may represent a more affected subset of the population with OPMD, particularly at younger ages because those who are pre-symptomatic or mildly symptomatic would have less reason to engage with MDC and the BIND survey.

### 4.1 Direct costs of OPMD

The direct costs of medical and assistive equipment for OPMD management were low relative to the indirect costs of OPMD. The need for supportive medical and assistive devices may be understated by our study due to demand suppression from financial factors. For example, low HRQoL was associated with higher out-of-pocket equipment costs, lower household income, worse greater financial toxicity and greater financial hardship, and those with OPMD had lower household incomes than the general population. In contrast, there was no relationship between HUI3 scores and visits with allied health professionals, however, accessibility of these services associated with access with private health insurance (odds ratio 2.45), which was in turn associated with paid employment status (odds ratio 4.18). Collectively, these findings suggest that medical equipment and healthcare support services may be generally less accessible to those who need these supports the most. Importantly, we did not capture overall use of medical and assistive devices by people with OPMD, only those associated with out-of-pocket costs, making the total cost likely substantially greater than that reported here.

### 4.2 Indirect costs of OPMD

Total productivity loss attributed to OPMD was 32 ± 27%, with presenteeism accounting for the majority (29 ± 27% vs. 4 ± 8% for absenteeism). The total productivity loss in OPMD is over 5-fold higher than the 5.9% calculated for 45-to 64-year-olds in the general Canadian population (68). Applying the total work loss calculation to absenteeism and presenteeism data reported for other chronic conditions (68) reveals that the total productivity loss in OPMD was comparable with stroke (28%), and higher than mood disorders (23%), heart disease (14%), cancer (13%), and back problems (13%). The reduced employment rate identified for OPMD compared to the age and sex-matched population (29.1% vs. 40.7%) has implications for long-term financial stability through reduced pensions and savings.

While non-work activity was the highest cost contribution for OPMD, it may be considered a conservative estimate. Our method to evaluate non-work activity imposed a cap that varied between $48.66 and $73.66 per day (sex- and age-dependent). These valuations are lower than those placed on unpaid housework in Canada by either the gross opportunity cost ($89.49 per day) or the net opportunity cost ($77.73 per day) (69). These valuations are also lower than the valuation placed on household service among individuals aged over 55 years in the United States, which ranged from $68.92 to $82.00 per day (70), after adjustment for purchasing power parity and inflation (71).

### 4.3 Limitations

Aspects of the study design (e.g., most participants were registered with MDC, survey completion was voluntary and uncompensated), may have introduced selection bias that could limit generalizability of the findings to the broader population with OPMD. Patient-reported costs might be subject to recall bias, misreporting, or inaccuracies. While there is also risk of misclassification bias, the characteristics of participants classified as having OPMD were consistent with the known epidemiology of OPMD in Canada. In particular, participants with OPMD were older on average than those with other NMDs in BIND, and a high proportion were from French-speaking regions. Nonetheless, some diagnostic misclassification cannot be excluded and may have affected our estimates. There may also be selection bias such that those with higher symptom burden may be more likely to engage with Muscular Dystrophy Canada and the BIND survey. This study was not based on a national OPMD registry but was part of the BIND NMD project (12) and therefore questionnaires were not specific to OPMD. Some potential cost-driving factors could therefore not be considered in analysis, such as ambulation, fatigue, disease severity (dysphagia, ptosis, limb weakness) and comorbidities. Absenteeism was framed as time taken off work. No aspect of the BIND study captured the lost earning capacity among those who chose to work fewer hours due to their NMD (e.g., choosing part time work over full time work to better manage NMD), and uncaptured reductions in working hours are likely to have substantial indirect cost to individuals with OPMD and society. However, this study is the largest health socioeconomic study in OPMD. These findings can inform the design of future prospective studies linking direct and indirect costs to health utilization data for the full impact of disease costs for the patient and health care system.

## 5 Conclusion

This study examined the cost of illness for a progressive, adult-onset muscle condition from the patient perspective. This examination of hidden and out-of-pocket expenses, sociodemographic disparities, and impact on HRQoL has revealed substantial direct and indirect burden imposed by OPMD on individuals and households and can inform health policy and evaluations of cost-effectiveness of emerging therapeutic interventions. The analytic framework and methods used in this study may be adapted to assess the economic burden of OPMD and other rare and chronic conditions in different healthcare and socioeconomic settings.

## Data Availability

The original contributions presented in the study are included in the article/Supplementary material, further inquiries can be directed to the corresponding author.

## Supplementary material

Supplementary Figure S1 Single attribute health utilities index mark 3 (HUI3) scores stratified by age and sex. No formal statistical comparisons were conducted due to low n and limited variability of scores within each attribute.

Supplementary Figure S2 The total patient-facing cost of oculopharyngeal muscular dystrophy in Canada as estimated using different age-cutoffs for mean age of onset of symptom burden. Values are 2026 Canadian dollars.
